# Research on Point Cloud Registering Method of Tunneling Roadway Based on 3D NDT-ICP Algorithm

**DOI:** 10.3390/s21134448

**Published:** 2021-06-29

**Authors:** Jianjian Yang, Chao Wang, Wenjie Luo, Yuchen Zhang, Boshen Chang, Miao Wu

**Affiliations:** School of Mechatronics and Information Engineering, China University of Mining and Technology, Beijing 100083, China; sqt1800402039@student.cumtb.edu.cn (C.W.); zqt2000402052@student.cumtb.edu.cn (W.L.); sqt2000402043@student.cumtb.edu.cn (Y.Z.); zqt1900401001g@student.cumtb.edu.cn (B.C.); wum@cumtb.edu.cn (M.W.)

**Keywords:** tunneling roadway, environmental modeling, point cloud registering, registering error

## Abstract

In order to meet the needs of intelligent perception of the driving environment, a point cloud registering method based on 3D NDT-ICP algorithm is proposed to improve the modeling accuracy of tunneling roadway environments. Firstly, Voxel Grid filtering method is used to preprocess the point cloud of tunneling roadways to maintain the overall structure of the point cloud and reduce the number of point clouds. After that, the 3D NDT algorithm is used to solve the coordinate transformation of the point cloud in the tunneling roadway and the cell resolution of the algorithm is optimized according to the environmental features of the tunneling roadway. Finally, a kd-tree is introduced into the ICP algorithm for point pair search, and the Gauss–Newton method is used to optimize the solution of nonlinear objective function of the algorithm to complete accurate registering of tunneling roadway point clouds. The experimental results show that the 3D NDT algorithm can meet the resolution requirement when the cell resolution is set to 0.5 m under the condition of processing the point cloud with the environmental features of tunneling roadways. At this time, the registering time is the shortest. Compared with the NDT algorithm, ICP algorithm and traditional 3D NDT-ICP algorithm, the registering speed of the 3D NDT-ICP algorithm proposed in this paper is obviously improved and the registering error is smaller.

## 1. Introduction

The environment of the tunneling face in coal mines is complex and the intelligence degree is low, making it difficult for tunneling equipment to perceive the work environment intelligently [1]. Environmental modeling for underground roadways is an effective way to solve the above problems. Sensors can be used to acquire the environmental point cloud data of tunneling roadways and establish the environmental point cloud map, which is convenient for guiding and carrying out the excavation and mining work [2,3]. However, due to the limitations of the special underground environment, the point cloud map generated directly has low accuracy and large errors in general. At present, improving the accuracy of the underground environment map of coal mines is the primary difficulty in the intelligent construction of the tunneling face [4].

Technologies such as 3D scanning and DT (Digital Twins) have received extensive attention in the industrial field. In terms of building inspection, the German Spacetec company developed the TS3 vehicle-mounted tunnel scanning system. The system adopts the principle of three-dimensional laser scanning measurement, which can perform crack detection, tunnel boundary detection and temperature collection [5]. The British Balfour Beatty Raily company (London, UK), developed the LaserFleX^TM^ system, which accurately detects the boundary of the tunnel based on the laser three-dimensional scanning method [6]. Columbia University in the United States used a three-dimensional laser scanner to collect data on the collapsed area and calculate changes in the amount of earthwork and changes in contour boundaries [7]. Shi Xinxiao et al. verified the feasibility of 3D laser technology in roadway modeling, which provides a more accurate digital model for digital mine construction [8]. Zhu Haibin et al. used 3D laser scanning technology to obtain 3D information of the roadway and establish a 3D digital model of the roadway, which improved the efficiency of surveying and mapping work in the roadway engineering [9]. In the protection of cultural relics, W. Neubauer Et al. used the Rigel 3D laser scanner to collect data on the Sphinx of Giza, provide data support for the protection and maintenance of the Sphinx and store a complete 3D digital model of the Sphinx [10,11]. In terms of digital cities, Vosselman et al. combined 3D laser scanning technology with 2D cadastral vector data, and extracted the height of houses through airborne laser scanning to build a 3D city model [12]. The above research methods all provide solutions for environmental modeling of tunneling roadways. However, in the process of generating the point cloud map of tunneling roadways, such solutions would be affected by the internal constraints of the roadway and cause large errors. Therefore, in order to further improve the accuracy of environmental modeling of tunneling roadways, it is also necessary to register the initial point cloud of tunneling roadways.

At present, the commonly used 3D point cloud registering algorithm is the Iterative Closest Points (ICP) [13]. The ICP algorithm basically meets the registering requirements of most 3D point clouds, but its running speed and accuracy mainly depend on the given initial transformation estimation, the size of the point cloud and the initial position accuracy [14]. Chen H et al. proposed a Two Step ICP (TICP) algorithm [15]. The method firstly uses the ICP algorithm for coarse registering on initial point cloud data, and then applies the obtained transformation matrix as initial transformation to the ICP algorithm again, which improves the registering efficiency. SI Choi et al. accelerated the ICP algorithm based on CUDA [16], but it is still difficult to meet the requirements of fast registering of actual point clouds. In order to overcome the above ICP algorithm problems, He et al. proposed a principal component analysis method for point cloud registration. This method mainly provides a good initial value for the ICP calculation by transforming the dimensions of the data. However, when there are many noise points in the point cloud, it is difficult for this algorithm to obtain a good initial value [17]. Yang et al. proposed a Scale-ICP algorithm based on seven-dimensional space iteration. Compared with the traditional ICP algorithm, this method has a certain degree of improvement in iterative speed and registration accuracy, but it still relies on the iterative process, which leads to the problem of slow convergence of the algorithm [18]. Sharp et al. proposed the ICPIF (Iterative Closest Points using Invariant Features) algorithm to improve the accuracy of finding point pairs [19]. In recent years, another registering algorithm proposed by Biber P and Strasser W, Normal Distribution Transform (NDT), has gradually attracted the attention of scholars [20]. The NDT algorithm uses standard optimization technology to determine the best registering of two point clouds [21]. In the registering process, the features of corresponding points are not used to calculate the registering of point clouds. For a large number of point cloud data, the registering speed is faster than ICP, but its registering accuracy is not as high as ICP. After that, relevant scholars started the fusion research of NDT and ICP algorithms, aiming at realizing fast and accurate registering of point clouds. Sobreira H compared the NDT and ICP algorithms, and added a new algorithm to PCL, which can improve the matching efficiency of the ICP algorithm [22]. Pang S fully tested the NDT and ICP algorithms through experiments, and the experimental results show that the NDT algorithm has better ability to deal with real adversity and higher computational efficiency [23]. Attia M uses a combination of NDT and ICP algorithms. The experimental results show that using the ICP algorithm to coarsely register the point cloud and then using the NDT algorithm for refinement is better than directly using the ICP algorithm for refinement [24]. Wang Qingshan et al. verified the reliability and accuracy of the combination of NDT and ICP algorithms in the campus environment [25]. Zhang Guiyang et al. used NDT and ICP algorithms [26], and their experiments verified that an NDT-ICP algorithm can improve the speed and accuracy of point cloud registering. Wang Yanming et al. applied an NDT-ICP point cloud registering algorithm on the basis of traditional robot 3D topography flexibility measurement data [27], which improves the speed and accuracy of robot topography flexibility measurement. Based on the above research, this paper optimizes and improves 3D NDT and ICP algorithms, and proposes a 3D NDT-ICP method suitable for rapid registering of 3D point clouds in tunneling roadways, which provides a technical scheme for environmental modeling of tunneling roadways.

## 2. Mathematical Analysis of Tunneling Roadway Environment

**Figure 1:**
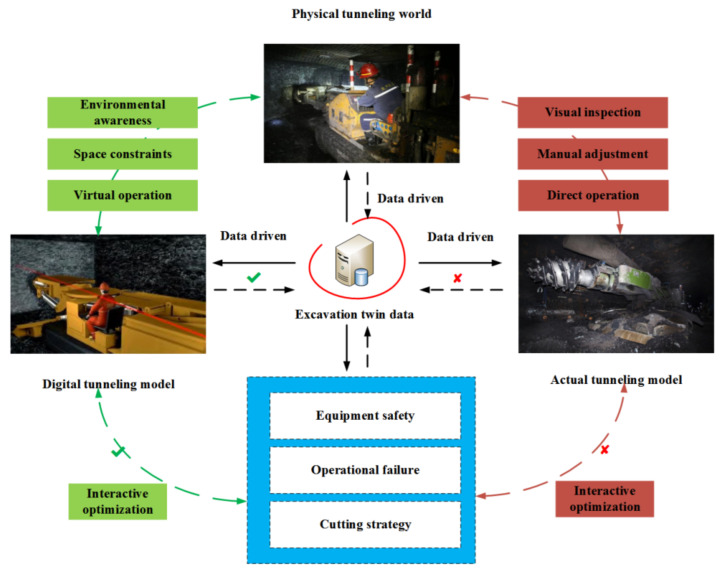
Digital twin conceptual model of tunneling face.

A tunneling roadway is an independent confined space under the coal mine. Environmental modeling and twin model construction are convenient to realize environmentally intelligent perceptions of a tunneling face and create a safe “man–machine–environment” interaction foundation, as shown in Figure 1.

### 2.1. Selection of Point Cloud Acquisition Method for Tunneling Roadways

**Figure 2:**
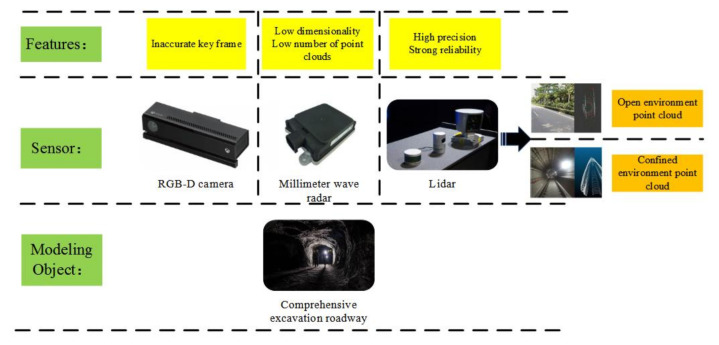
Comparison of point cloud acquisition methods.

At present, the research on twin models of tunneling roadway environments is continuously developing [28]. According to sensor selection, the commonly used methods for obtaining point clouds of tunneling roadways are mainly divided based on vision camera, based on millimeter wave radar, based on laser radar or laser scanner, etc. Figure 2 shows the use features of the existing point cloud acquisition methods in the tunneling environment.

As can be seen from Figure 2, Lidar has shown unique advantages in tunneling: high data accuracy and strong reliability. At the same time, Lidar obtains less point cloud data in an open and open environment, and obtains more point clouds in a confined space such as roadways, which provides good data support for subsequent tunneling roadway environment modeling.

### 2.2. Feature Analysis of Several Point Cloud Data in Tunneling Roadways

**Figure 3:**
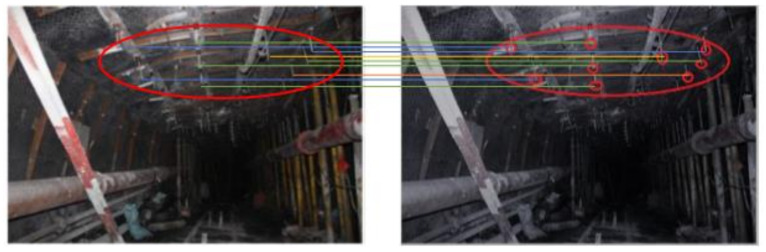
“Anchor protection point cloud” roadway.

In tunneling roadways, most of the point cloud information can be scanned directly by Lidar, but some special point cloud information belongs to “discrete point cloud”, which is easily confused with wrong point cloud, such as “anchor protection point cloud” (point cloud composed of anchor end points), as shown in the red circle in Figure 3. There are differences between the distribution of this kind of point cloud and the overall point cloud in the roadway, which is easy to be regarded as wrong point cloud and screened out by filtering algorithm in the preprocessing process.

Firstly, the point cloud of a tunneling roadway is defined as R (Roadway Point Cloud). As the tunneling roadway needs to be supported by an anchor rod, the end points of the anchor rod form a group of relatively discrete point clouds $R_{ar}$ (i.e., anchor protection point clouds) inside the roadway. In the point cloud R, a neighborhood U is selected for any point in the anchor protection point cloud $R_{ar}$. In the neighborhood U, the mean and variance of the mean distance between the point and all point clouds are calculated.

The points in the anchor protection point cloud $R_{ar}$ mainly have the following mathematical features:

(1) $$\left|R_{ar}(x,y,z)-\mu \right|\;\geq \;\sigma F$$

In Formula (1), F depends on the number of points in the neighborhood.

### 2.3. Environmental Constraints for Accurate Modeling of Tunneling Roadways

**Figure 4:**
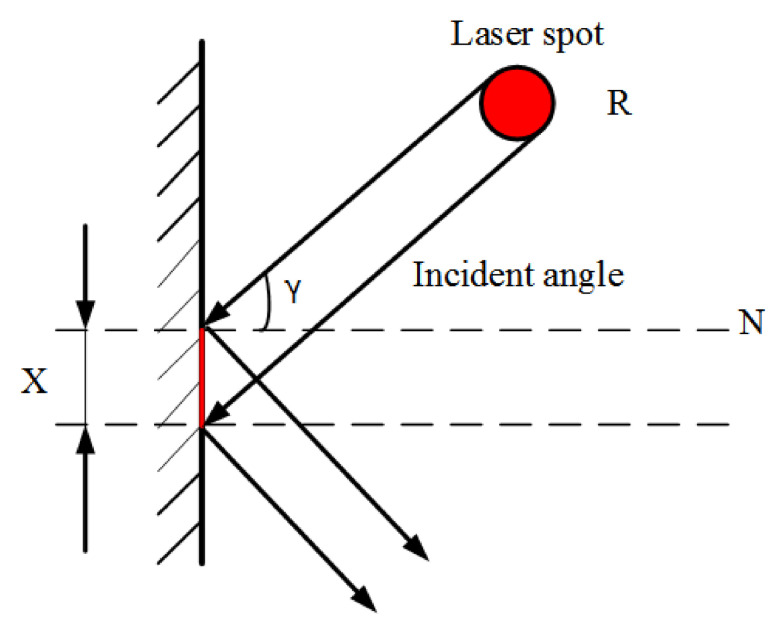
Influence of incident angle on long axis of light spot.

The non-contact measurement accuracy of Lidar in a confined space is mainly affected by the size of the light spot. In the process of scanning and measuring tunneling roadways, the laser beam irradiates the surface of the measuring object to form a light spot, and the radar laser point is theoretically the central position of the light spot. However, the laser point may in fact be located at any position of the spot, so there is an influence of the spot size on the measurement accuracy of the laser point. There are many factors that affect the spot size. Among the many factors, scanning features (incident angle and distance) have the greatest effect [29]. When the incident angle and measurement distance change, the spot area would be affected, which affects the laser point measurement accuracy of Lidar, as shown in Figure 4. M. Bitenc showed Equation (2) for the change in the long axis X of the elliptical spot [30].

(2) $$X=\frac{SH}{\cos\gamma }(0^{{}^{\circ}}\leq \gamma ≺90^{{}^{\circ}})$$

In Equation (2), S is the measurement distance, H is the laser beam linewidth, γ is the incident angle and R is the spot diameter.

**Figure 5:**
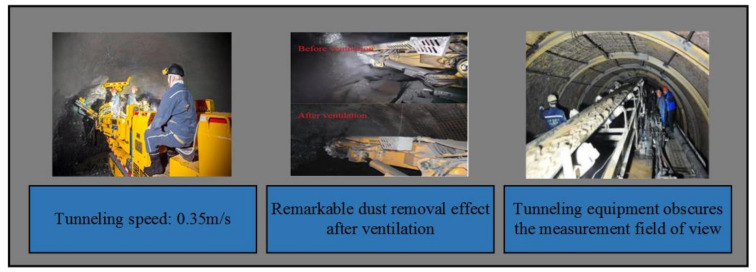
External features of a tunneling roadway.

As shown in Figure 5, combined with the tunneling roadway environment, it can be seen that Lidar has good applicability underground, but it is affected by the inevitable constraints of the roadway environment. For example, ① meteorological conditions such as temperature, air pressure and humidity inside the roadway would affect the atmospheric refractive index, and thus affect the distance measurement accuracy of Lidar. ② The temperature gradient and atmospheric vibration in the direction of the light path in the roadway would affect the direction of the light, and then increase the angle measurement error of Lidar. ③ The roadway equipment body shields part of the laser radar measurement field of vision. As a result, the tunnel point cloud information generated by a single scanning of Lidar is not perfect, and random errors and some outliers are easily superimposed—i.e., the wrong point cloud $R_{e}$ is generated.

Therefore, it is necessary to use a point cloud registering method to splice laser point clouds from different viewing angles to improve the modeling results of roadway environment. However, the wrong point cloud $R_{e}$ generated in the generation process of laser point cloud would have certain influence on the registering accuracy and efficiency of tunneling roadway point clouds. When the number of points in the wrong point cloud $R_{e}$ is large, it would interfere with the search of corresponding points of point clouds and generate wrong registering point pairs, which can easily cause large deviation in coarse registering of point clouds and local convergence in fine registering of point clouds. Therefore, it is necessary to preprocess the initial point cloud of a tunneling roadway and design an appropriate registering method.

## 3. Research on Environmental Modeling Method of Tunneling Roadways

### 3.1. Pretreatment of Point Cloud Data in Tunneling Roadways

At present, the filtering methods for laser scattered point clouds can be generally divided into two types. The first type is to convert the point cloud model into a grid model for filtering processing; the second type is to filter directly by a filtering method. Considering that anchor protection point cloud $R_{ar}$ is easily confused with wrong point cloud $R_{e}$ in tunneling roadways, it would screen out some point clouds of a tunneling roadway and destroy the overall structure of the point cloud of the tunneling roadway by preprocessing the initial point cloud of the tunneling roadway by the second type of direct filtering method, so the first type of filtering method is selected to preprocess the initial point cloud of the tunneling roadway. After the point cloud model of the tunneling roadway is converted into a grid model, the point cloud data in each grid is filtered and screened to ensure the integrity of the point cloud structure and remove a large number of unnecessary point clouds. In this paper, a Voxel Grid filtering method is used to preprocess point cloud data. The specific mathematical derivation is as follows:

(1) According to the coordinate set of point cloud data, the maximum values $x_{\max}$, $y_{\max}$ and $z_{\max}$ the minimum values $x_{\min}$, $y_{\min}$ and $z_{\min}$ on the three coordinate axes of X, Y and Z are obtained.

(2) According to the maximum and minimum values on the three coordinate axes of X, Y and Z, the side length $l_{x}$, $l_{y}$ and $l_{z}$ of the minimum bounding box of the point cloud are obtained.

(3) $$\left\{\begin{array}{c} l_{x}=x_{\max}-x_{\min}\\ l_{y}=y_{\max}-y_{\min}\\ l_{z}=z_{\max}-z_{\min} \end{array}\right.$$

(3) Set the side length of the voxel small grid cell and divide the X, Y and Z coordinate axes into M, N and L parts equally, then the minimum bounding box is divided into M*N*L voxel small grid, sum=M*N*L.

(4) $$\begin{array}{ccc} M=\left\lfloor \frac{l_{x}}{cell}\right\rfloor & N=\left\lfloor \frac{l_{y}}{cell}\right\rfloor & L=\left\lfloor \frac{l_{z}}{cell}\right\rfloor \end{array}$$

In Equation (4), $\left\lfloor •\right\rfloor$ means rounding down and sum is the total number of voxel small grids.

(4) Number the small grid of each voxel—the numbers are (i,j,k)—and determine the voxel cell to which each data point belongs.

(5) $$\begin{array}{ccc} i=\left\lfloor \frac{x_{i}-x_{\min}}{cell}\right\rfloor & j=\left\lfloor \frac{y_{i}-y_{\min}}{cell}\right\rfloor & k=\left\lfloor \frac{z_{i}-z_{\min}}{cell}\right\rfloor \end{array}$$

(5) Carry out point cloud reduction filtering. Calculate the center of gravity of each voxel small grid, and replace all points in the voxel small grid with the center of gravity. If the center of gravity does not exist, all points in the small grid are replaced by the data point closest to the center of gravity.

(6) $$c_{ijk}=\frac{1}{k}\sum_{i=1}^{k}p_{i}$$

where $c_{ijk}$, $p_{i}$ and k are the center of gravity, data points and points of voxel small grids, respectively.

### 3.2. Accurate Registering Method of Point Cloud in Tunneling Roadways

After preprocessing the point cloud data, four groups of experimental point clouds are obtained, one of which is recorded as the reference point cloud M(x,y,z) and the other as the point cloud to be registered N(x,y,z). The two have overlapping areas, O(x,y,z) and O=M∩N. In order to reduce the influence of roadway internal environment constraints, reduce the registering error of tunneling roadway point clouds and improve the registering speed, this paper integrates the advantages of NDT and ICP algorithms, and proposes a registering method of tunneling roadway point clouds based on a 3D NDT-ICP algorithm.

**Figure 6:**
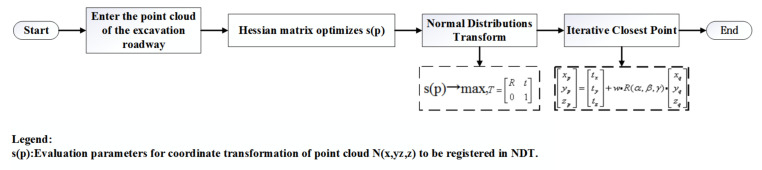
Flow of a traditional 3D NDT-ICP algorithm.

Most of the traditional 3D NDT-ICP algorithms use two-step combination registering. First, the 3D NDT algorithm is used to correct the initial input point cloud pose, and then the ICP algorithm is used to register the point cloud after pose correction. The specific process is shown in Figure 6.

In the traditional 3D NDT-ICP algorithm, the 3D NDT algorithm first performs coarse registration on the point cloud that needs to be registered, corrects the initial position of the point cloud to be registered and makes it approach the reference point cloud. Then, the ICP algorithm is used for fine registration, and the point cloud to be registered after the 3D NDT algorithm is registered. In the traditional two-step 3D NDT-ICP algorithm, the project files need to be compiled separately, a total of two times, the efficiency is low and the registration process is not coherent.

**Figure 7:**
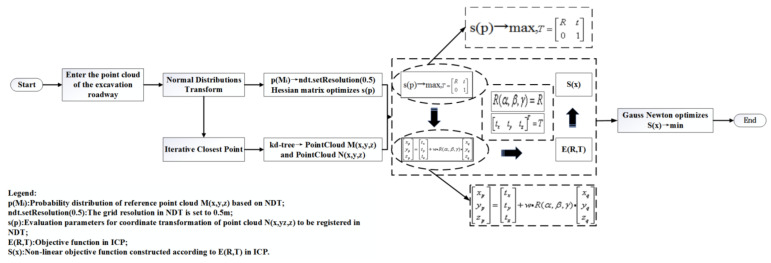
The 3D NDT-ICP algorithm flow.

As shown in Figure 7, the flow of the 3D NDT-ICP algorithm proposed in this paper is firstly optimized according to the environmental features of tunneling roadways, and then the point cloud coordinate transformation matrix solved by the 3D NDT algorithm is taken as the initial matrix of the ICP algorithm to ensure the consistency of registering. Then, a kd-tree is used for point pair search in the registering process of ICP algorithm to improve the registering speed of point cloud. Finally, the Gauss–Newton method is used to iteratively optimize the objective function of ICP algorithm to solve the optimal coordinate transformation parameters between point clouds and complete the accurate registering of point clouds.

#### 3.2.1. Solution of Point Cloud Coordinate Transformation Parameters in Tunneling Roadways

Firstly, the reference point cloud M(x,y,z) data sample is evenly divided into several regular 3D voxel units with the same size by a 3D NDT algorithm, and then the probability distribution of each 3D point cloud position in the 3D voxel unit is expressed by normal distribution; the expression is shown in Equation (7). The points of the corresponding point pair in the reference point cloud M are denoted as $M_{i}$, $M_{i}=(x_{M},y_{M},z_{M}),M_{i}\in M,i=1,2,3,\ldots$, and the points in the point cloud N to be registered are denoted as $N_{i}$, $N_{i}=(x_{N},y_{N},z_{N}),N_{i}\in N,i=1,2,3,\ldots$.

(7) $$p(M_{i})=\frac{1}{c}\exp [-\frac{{\left(M_{i}-q\right)}^{T}C^{-1}(M_{i}-q)}{2}]$$

The parameter C is the covariance matrix of the 3D point clouds in each voxel unit and q is the mean value of the 3D point clouds in each voxel cell. The parameter c is a constant. Specific definitions of q and C are shown in Equations (8) and (9).

(8) $$q=\frac{1}{n}\sum_{i=1}^{n}M_{i}$$

(9) $$C=\frac{1}{n-1}\sum_{i=1}^{n}\left(M_{i}-q\right){\left(M_{i}-q\right)}^{T}$$

(10) c=(2π)D2C

In Equation (10), D represents the number of dimensions. $M_{i}(i=1,......,n)$ represents all 3D point cloud data in voxel cells.

Equation (11) initializes the transformation parameters for the point cloud M and N:

(11) $$T= [\begin{array}{cc} R & t\\ 0 & 1 \end{array}]$$

According to coordinate transformation parameters, each point cloud sample of the point cloud to be registered N(x,y,z) is mapped into a reference point cloud M(x,y,z) data sample coordinate system. The mapped point cloud is recorded as $N^{\prime }$, the probability distribution of each 3D point mapping in $N^{\prime }$ is summed and the coordinate transformation parameters are evaluated:

(12) $$s(p)=\sum_{i}\exp [-\frac{{\left(N_{i}{}^{\prime }-q_{i}\right)}^{T}C^{-1}\left(N_{i}{}^{\prime }-q_{i}\right)}{2}]$$

Through a Hessian matrix method, s(p) is optimized to maximize the value of s(p). The problem of solving the optimal transformation of the matrix is regarded as the process of minimizing s(p). The realization of this process is solved by the Newton method and the Hessian matrix. Let f=s(p); in order to minimize the function f, the following equations must be processed for each operation:

(13) HΔp=−g

In Equation (13), g is the transposed gradient of f, and the specific elements can be expressed as:

(14) $$g_{i}=\frac{\partial f}{\partial p_{i}}$$

H is the Hessian matrix of f, and its elements can be expressed as:

(15) $$H_{ij}=\frac{\partial^{2}f}{\partial p_{i}\partial p_{j}}$$

After obtaining Δp, we can update Δp with the following formula:

(16) p←p+Δp

#### 3.2.2. Transfer of Point Cloud Coordinate Transformation Parameters in Tunneling Roadways

Any point $P(x_{p},y_{p},z_{p})$ in the reference point cloud is M, and any point $Q(x_{q},y_{q},z_{q})$ in the point cloud is to be registered N. Meanwhile, P and Q satisfy P∈(M∩O) and Q∈(N∩O), and the external conversion relationship of the two independent 3D coordinate systems is further described by using the seven-parameter spatial similarity transformation model through the ICP algorithm [31], as shown in Formula (17).

(17) $$[\begin{array}{c} x_{p}\\ y_{p}\\ z_{p} \end{array}]= [\begin{array}{c} t_{x}\\ t_{y}\\ t_{z} \end{array}]+w•R(\alpha ,\beta ,\gamma )• [\begin{array}{c} x_{q}\\ y_{q}\\ z_{q} \end{array}]$$

where $t_{x}$, $t_{y}$ and $t_{z}$ are the three components along the coordinate axis direction, α, β and γ, and are the three angle parameters rotating around the coordinate axis; w is the scale transformation factor between coordinate systems, which generally defaults to 1. Then, the final result of Equation (11) is passed to the ICP algorithm to initialize the rotation matrix and translation matrix in Equation (17).

(18) $$\left\{\begin{array}{c} R(\alpha ,\beta ,\gamma )=R\\ { [\begin{array}{ccc} t_{x} & t_{y} & t_{z} \end{array}]}^{T}=t \end{array}\right.$$

In order to speed up ICP registering, this paper uses a kd-tree to retrieve roadway point cloud in the ICP algorithm part. This data search method is widely used in the field of point cloud research, so it is not listed as the focus of introduction. Assuming that the two groups of point clouds in the tunneling roadway jointly generate m groups of corresponding point pairs, when Equation (19) obtains the optimal solution, the two groups of point clouds complete the solution of the spatial position conversion relationship.

(19) $$E(R,T)=\frac{1}{m}\sum_{i=1}^{m}{\left\|M_{i}-(RN_{i}+T)\right\|}^{2}$$

In order to facilitate the derivation of the subsequent iterative optimization equation, in Equation (19) ${\left\|M_{i}-(RN_{i}+T)\right\|}^{2}=d^{2}=D$, d represents the Euclidean distance between $M_{i}$ and $N_{i}$, as shown in Equation (20):

(20) $$d(M_{i},N_{i})\;=\;\left|M_{i}-N_{i}\right|\;=\;\sqrt{{\left(x_{M}-x_{N}\right)}^{2}+{\left(y_{M}-y_{N}\right)}^{2}+{\left(z_{M}-z_{N}\right)}^{2}}$$

#### 3.2.3. Iterative Optimization of Point Cloud Coordinate Transformation Parameters in Tunneling Roadways

The objective function E(R,T) in Equation (19) needs to be optimized. That is, Equation (23) is minimized. Due to the nonlinearity of the objective function, the estimated values of parameters cannot be obtained by solving the extreme values of linear least squares and other multivariable functions, so complex optimization algorithms are needed to solve this problem. In this paper, the Gauss–Newton method is used to optimize the objective function in ICP algorithm, so as to avoid local convergence of tunneling roadway point clouds in the process of precise registering.

The general form of the nonlinear system model is:

(21) $$\min S(x)=f^{T}(x)f(x)={\left\|f(x)\right\|}^{2}$$

Among them, $f(x)={\left(f_{1}(x),f_{2}(x),\ldots ,f_{m}(x)\right)}^{T}$,$x={\left(x_{1},x_{2},\ldots ,x_{n}\right)}^{T}$.

The sum of squares of Euclidean distances at a certain point between two groups of point clouds in the roadway before and after registering is:

(22) $$D=d^{2}(M_{i},N_{i})={\left|M_{i}-N_{i}\right|}^{2}={\left(x_{M}-x_{N}\right)}^{2}+{\left(y_{M}-y_{N}\right)}^{2}+{\left(z_{M}-z_{N}\right)}^{2}$$

We can substitute Equation (21) into Equation (22) for iterative operation processing:

(23) $$\min S(x)=f^{T}(x)f(x)={\left\|f(x)\right\|}^{2}=\sum D$$

Among them, $f(x)={\left(f_{1}(x),f_{2}(x),\ldots ,f_{m}(x)\right)}^{T}$, $x={\left(t_{x},t_{y},t_{z},w,\alpha ,\beta ,\gamma \right)}^{T}$.

If the point of the k iteration is $x^{k}$, it can be seen from the Taylor expansion equation that:

(24) $$f_{i}(x)\approx f_{i}(x^{k})+\nabla f_{i}{\left(x^{k}\right)}^{T}(x-x_{k}),(i=1,2,\ldots ,m)$$

(25) $$f(x)=\sum_{i=1}^{m}f_{i}(x)\approx f(x^{k})+A(x^{k})(x-x_{k})$$

$A(x^{k})$ is a multidimensional first-order partial derivative matrix, namely a Jacobian matrix:

(26) $$A(x^{k})={ [\begin{array}{ccc} \frac{\partial f_{1}}{\partial t_{x}} & \cdots & \frac{\partial f_{1}}{\partial \gamma }\\ \vdots & \vdots & \vdots \\ \frac{\partial f_{m}}{\partial t_{x}} & \cdots & \frac{\partial f_{m}}{\partial \gamma } \end{array}]}_{x=x^{k}}={\left(\frac{\partial f_{i}(x^{k})}{\partial x_{j}}\right)}_{m\times n},n=7$$

In the above equation, $x_{j}={\left(t_{x},t_{y},t_{z},w,\alpha ,\beta ,\gamma \right)}^{T}$.

With $A_{k}=A(x^{k})$, the Equation (25) is combined and brought into the Equation (23), the following results can be obtained:

(27) $$S(x)\approx {\left\|f(x^{k})+A_{k}(x-x^{k})\right\|}^{2}={ [A_{k}d^{k}+f(x^{k})]}^{T} [A_{k}d^{k}+f(x^{k})]$$

Among them, $d^{k}=x-x^{k}$. For the minimum problem of minS(x), the least square method can obtain:

(28) $$A_{k}{}^{T}A_{k}d^{k}=-A_{k}{}^{T}f(x^{k})$$

when $A_{k}{}^{T}A_{k}$ is reversible, there are:

(29) $$d^{k}=-{\left(A_{k}{}^{T}A_{k}\right)}^{-1}A_{k}{}^{T}f(x^{k})$$

Because $d^{k}=x-x^{k}$, when $x^{k+1}=x=x^{k}+d^{k}$, in combination with Equation (29), there is an iterative equation:

(30) $$x^{k+1}=x=x^{k}+d^{k}=x^{k}-{\left(A_{k}{}^{T}A_{k}\right)}^{-1}A_{k}{}^{T}f(x^{k})$$

Because $\nabla S(x)=2\sum_{i=1}^{m}f_{i}(x)\nabla f_{i}(x)=2A^{T}(x)f(x)$, when $H_{k}=2A_{k}{}^{T}A_{k}$, then $H_{k}$ is the Hessian matrix of minS(x) at point $x^{k}$.

(31) $$H_{k}d^{k}=2A_{k}{}^{T}A_{k}d^{k}=-2A_{k}{}^{T}f(x^{k})=-\nabla S(x^{k})$$

In sum, it can be concluded that $d^{k}=-H_{k}{}^{-1}\nabla S(x^{k})=-A_{k}{}^{T}f(x^{k})$ is the Gauss–Newton equation and $x^{k+1}=x=x^{k}+d^{k}=x^{k}-H_{k}{}^{-1}\nabla S(x^{k})$ can be called the Gauss–Newton direction.

## 4. Experimental Verification

In the experiment, the operating system of the upper computer is Windows 10, the running memory is 8 G, and the software is Visual Studio 2017.

### 4.1. Point Cloud Data Preprocessing Experiment

**Figure 8:**
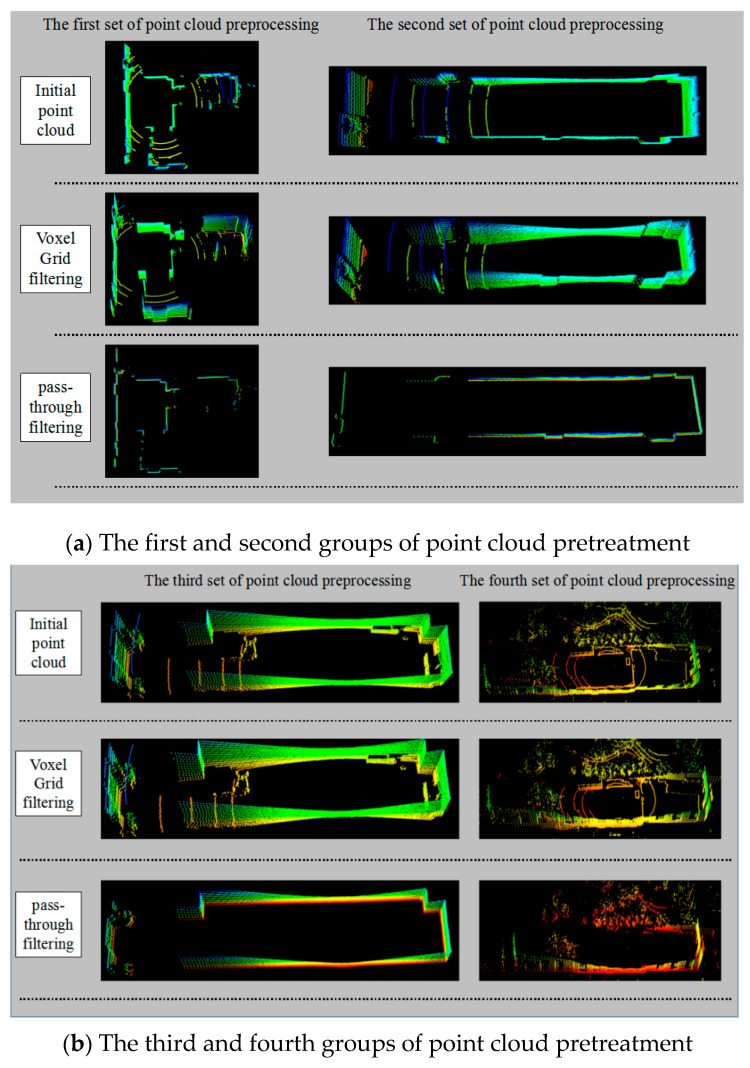
Point cloud data pretreatment.

First, make four sets of PCD files that simulate the point cloud of mine tunnels:

① (test1-a.pcd, test1-b.pcd), ② (test2-a.pcd, test2-b.pcd), ③ (test3-a.pcd, test3-b.pcd) and ④ (test4-a.pcd, test4-b.pcd). ① test1-a.pcd is the reference point cloud, test1-b.pcd is the point cloud to be registered; ② test2-a.pcd is the reference point cloud, and test2-b.pcd is the point cloud to be registered; ③ test3-a.pcd is the reference point cloud, test3-b.pcd is the point cloud to be registered; ④ test4-a.pcd is the reference point cloud, and test4-b.pcd is the point cloud to be registered. Then, the Voxel Grid filtering algorithm and pass-through filtering algorithm are used to preprocess the four groups of laser point clouds ①, ②, ③ and ④ respectively. Since the reference point cloud and the point cloud to be registered are essentially the same, this paper preprocesses the reference point cloud in the four groups of point clouds. The experimental results are shown in Figure 8, and the experimental data are shown in Table 1.

As can be seen from Table 1, the pass-through filtering reduces the number of laser point clouds more than the Voxel Grid filtering method. However, combined with Figure 8a,b, it can be seen that the pass-through filtering can easily destroy the point cloud structure of tunneling roadways, which results in a loss of integrity, while the Voxel Grid filtering method reduces the number of point clouds while ensuring the structural integrity of point clouds. It is more suitable for preprocessing point cloud data of tunneling roadways. The four groups of laser point clouds filtered by the Voxel Grid algorithm are recorded as experimental point clouds for subsequent experimental research.

### 4.2. Parameter Optimization Experiment of NDT Algorithm

**Figure 9:**
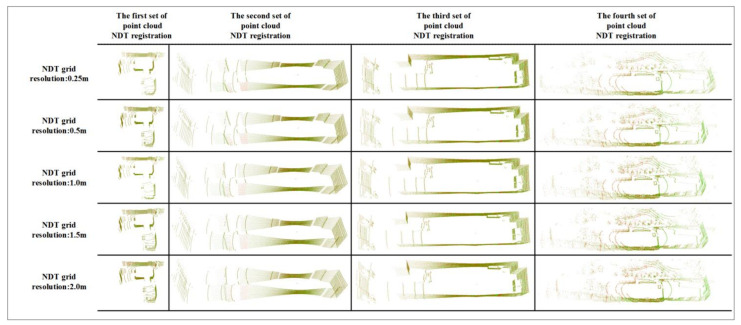
NDT registering results.

**Figure 10:**
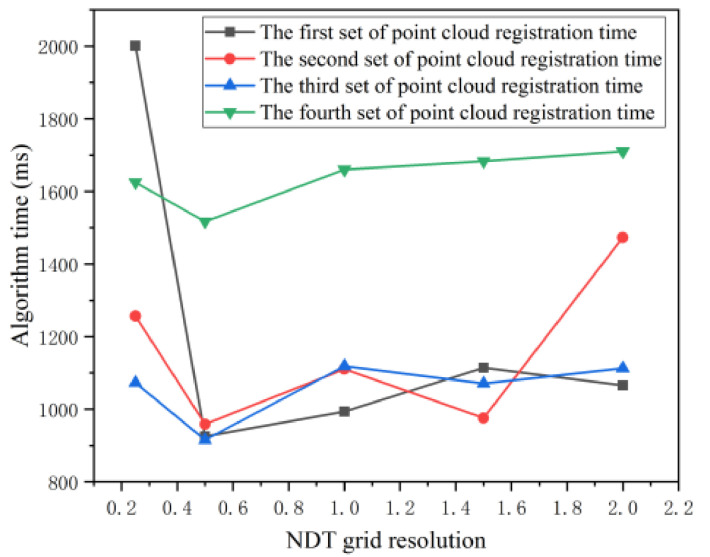
NDT registration time.

Influenced by Equation (7), the size of cells in 3D NDT algorithm affects the registering accuracy. Previously, the scholar Magnusson made a systematic comment on the cell size setting in 3D NDT method. When the cell setting is too large, it cannot well represent the features of point cloud. However, when the cell setting is too small, it is vulnerable to the noise of radar scanning equipment and may not be able to calculate the Gaussian distribution due to insufficient data in the cell. At the same time, Magnusson concluded through system experiments that the cell resolution of a 3D NDT algorithm is usually between 0.5 m and 2 m [32], which is ideal for laser scanning equipment. Combined with the above conclusions, in order to ensure that the cell resolution interval is sufficient, this paper chooses to set the cell resolution of the 3D NDT algorithm to 0.25 m, 0.5 m, 1 m, 1.5 m and 2 m. Based on four groups of experimental point clouds, experiments are carried out to provide the basis for selecting algorithm parameters for tunneling roadway environment modeling. The experimental results are shown in Figure 9, and the algorithm registration time is shown in Figure 10.

In Figure 9, the green display part is the reference point cloud, and the red display part is the point cloud to be registered. When designing the 3D NDT-ICP algorithm, this article aims to quickly obtain high-precision registration point clouds. Since the accuracy of the NDT algorithm has little effect on the overall 3D NDT-ICP algorithm in this paper, this paper focuses on the time spent by the NDT algorithm. It can be seen from Figure 10 that when using the 3D NDT algorithm to register the point cloud of the tunnel and roadway, setting the pixel resolution to 0.5 m can reduce the time spent by the algorithm to the shortest.

### 4.3. Comparative Experiment of Point Cloud Registering Algorithms

**Figure 11:**
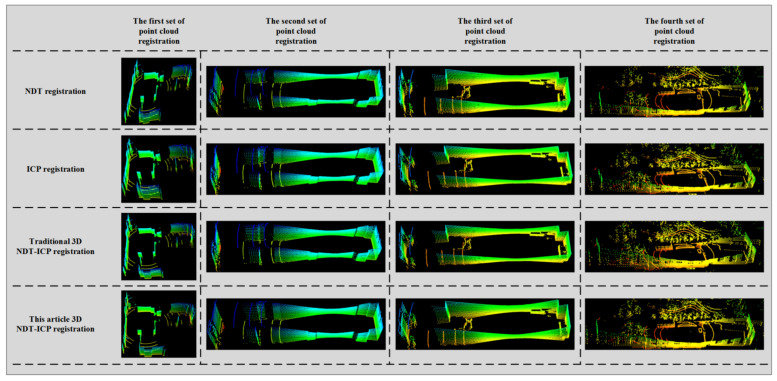
Final result of experimental point cloud registration.

In order to verify the rapidity and accuracy of the algorithm in this paper, this section carries out a point cloud registering comparative experiment. The initial iteration number of each algorithm is set to 100, and the initial grid resolution of the NDT algorithm is 1.0 m. The NDT algorithm, ICP algorithm, traditional 3D NDT-ICP algorithm and the 3D NDT-ICP algorithm proposed in this paper are respectively used to register the experimental point cloud. The registering results are shown in Figure 11.

It can be seen from Figure 11 that there is a certain error in the registration result of the NDT algorithm, and the precision of the point cloud registration is not high. The ICP algorithm has better registration results, and the point cloud precision is improved compared to the NDT algorithm. The traditional 3D NDT-ICP algorithm and the 3D NDT-ICP algorithm proposed in this paper have a good registration effect, and the point cloud outline is clear. Compared with the NDT algorithm, ICP algorithm and traditional 3D NDT-INDT algorithm, the 3D NDT-ICP algorithm proposed in this paper has better registration effect and higher precision.

**Figure 12:**
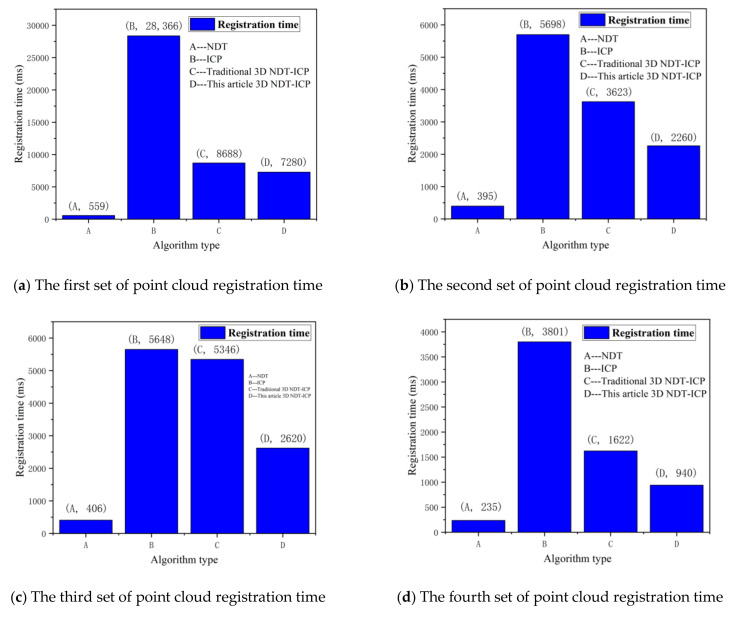
Point cloud registration time.

As can be seen from Figure 12, when registering the experimental point cloud, the NDT algorithm takes the shortest time, followed by the 3D NDT-ICP algorithm proposed in this paper, which takes shorter time than the traditional 3D NDT-ICP algorithm, and the ICP algorithm takes the longest time.

Because it is difficult to accurately judge the registration effect of the four algorithms through pictures, this paper introduces the root mean square error to judge the point cloud registration error. The registration effect of the algorithm is comprehensively evaluated by comparing the point cloud X, Y and Z three-axis root mean square error. The root mean square error calculation is shown in Formula (32).

(32) $$RMSE=\sqrt{\frac{\sum_{i=1}^{n}{\left(X_{i}-\hat{X_{i}}\right)}^{2}}{n}}$$

**Figure 13:**
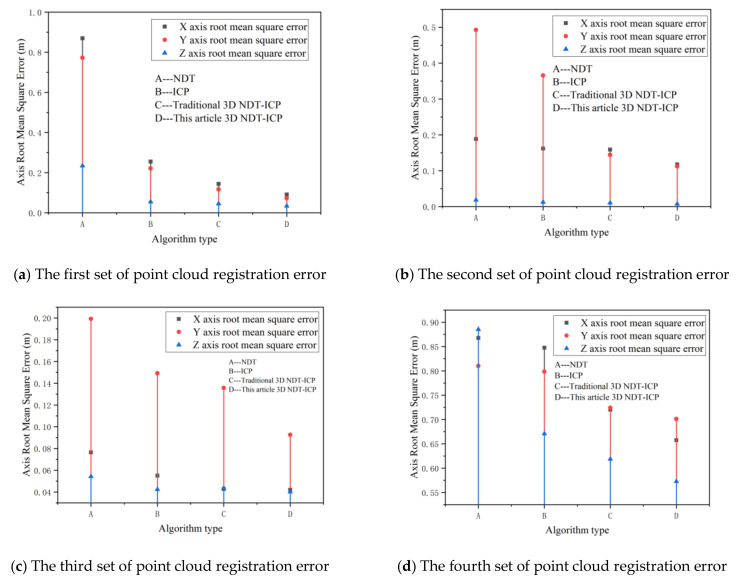
Point cloud registration error.

In Formula (32), n is the number of corresponding point pairs in the point cloud, $X_{i}$ is the Euclidean distance between the corresponding points after point cloud registration, and $\hat{X_{i}}$ is the true value of the Euclidean distance between the corresponding points. Under absolutely ideal conditions, the corresponding points are fully registered and the distance is zero. Therefore, the value of $\hat{X_{i}}$ here is 0. Figure 13 shows X, Y and Z three-axis root mean square error after the four sets of experimental point clouds are registered.

As can be seen from Figure 13, when registering the experimental point cloud, the 3D NDT-ICP algorithm proposed in this paper has the smallest registering error compared with the NDT algorithm, ICP algorithm and traditional 3D NDT-ICP algorithm.

Combined with Figure 12 and Figure 13, it can be seen that the 3D NDT-ICP algorithm proposed in this paper reduces the registering error of tunneling roadway point clouds and saves the registering time of point clouds when the experimental point clouds are matched.

**Figure 14:**
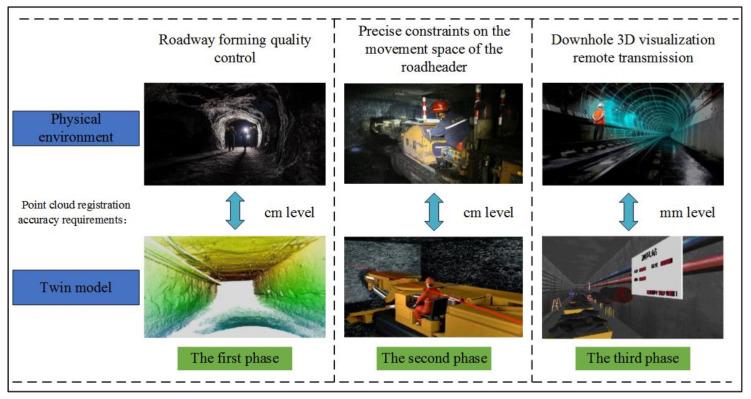
Accuracy requirements of an underground tunneling face.

As shown in Figure 14, the requirements for point cloud registering accuracy in different stages of the tunneling face are shown. Combined with Figure 13, it can be seen that the algorithm proposed in this paper meets the requirements of the quality control of tunneling roadways forming in the first stage and the constraint accuracy of roadheader motion space in the second stage. It is beneficial for the high-efficiency registration of large-scale point cloud data of tunneling roadways, and enhances the real-time and accuracy of the construction of twin models of tunneling face environment. Subsequent research is still needed to further improve the accuracy of point cloud models of tunneling roadways.

## 5. Conclusions

In this paper, a point cloud registering method for tunneling roadways based on 3D NDT-ICP algorithm is proposed, which is used to improve the modeling accuracy of the tunneling roadway environment and meet the requirements for an intelligent perception of tunneling environments. The main conclusions are as follows:

(1) In this paper, a Voxel Grid filtering method is used to preprocess the point cloud data of tunneling roadways, which can ensure the integrity of the point cloud structure of a tunneling roadway and reduce the number of point clouds, thus laying a foundation for the subsequent point cloud registering.

(2) Based on the environmental features of tunneling roadways, this paper optimizes the parameters of an NDT algorithm. The coordinate transformation parameters obtained by the NDT algorithm are used to initialize the seven-parameter coordinate transformation matrix of ICP algorithm, a kd-tree is introduced into ICP algorithm to search point pairs and the Gauss–Newton method is used to optimize and solve the objective function, thus realizing fast and accurate registering of tunneling roadway point clouds.

(3) Experiments show that the algorithm in this paper has a good effect on point cloud registering with tunneling tunnel environment features. However, the research object in this paper has certain limitations, and more cutting-edge and high-precision point cloud registration should be studied in follow-up algorithms.

## Figures and Tables

**Table 1:** Experimental data of laser point cloud pretreatment.

Point Cloud Group	First	Second	Third	Fourth
Number of initial point clouds	28,146	29,113	29,030	26,619
Number of point clouds after Voxel Grid filtering (unit: piece)	16,197	11,788	14,136	19,664
Number of point clouds after pass-through filtering (unit: piece)	10,486	7909	11,482	10,740
Point cloud data reduction/% (Voxel Grid filtering)	42.45	59.51	51.31	26.13
Point cloud data reduction/% (pass-through filtering)	62.74	72.83	60.45	59.65

## Data Availability

In this paper, a point cloud in kitti data provided by Karlsruhr Institute of Technology and Toyota Institute of Technology, and three corridors point cloud in the Science and Technology Building of China University of Mining and Technology (Beijing, China) are used.

## References

[1] Shirong G., Fan Z., Shibo W., Zhongbin W. (2020). Digital twin for smart coal mining workface: Technological frame and construction. J. China Coal Soc..

[2] Jianjian Y., Qiang Z., Miao W., Chao W., Boshen C., Xiaolin W., Shirong G. (2020). Research progress of autonomous perception and control technology for intelligent heading. J. China Coal Soc..

[3] Feng W., Jialin X., Jianlin X., Li Z., Guo J. (2016). Mechanisms influencing the lateral roof roadway deformation by mining-induced fault population activation: A case study. Int. J. Oil Gas Coal Technol..

[4] Wang G., Xu Y., Meng X., Fan J., Wu Q., Ren H., Pang Y., Du Y., Zhao G., Li M. (2020). Specification, classification and grading evaluation index for smart long wall mining face. J. China Coal Soc..

[5] Marino F., Distante A., Mazzeo P.L., Stella E. (2007). A real-time visual inspection system for railway maintenance: Automatic hexagonal-headed bolts detection. IEEE Trans. Syst. Man Cybern. Part C.

[6] Altuntas C. (2014). The Effect of Point Density on the Registration Accuracy of a Terrestrial Laser Scanning Dataset. Lasers in Engineering.

[7] Makadia A., Patterson A., Daniilidis K. Fully automatic registration of 3D point clouds. Proceedings of the IEEE Computer Society Conference on Computer Vision and Pattern Recognition (CVPR’06). IEEE.

[8] Xinxiao S., Jian W., Lei W., Li Z., Fu H. (2019). Three-dimensional Modelling of Mine Laneways Under Point Cloud Data. Remote Sens. Inf..

[9] Haibin Z. (2019). Application of 3D laser scanning technology in sinking and driving engineering. China Coal.

[10] Neubauer W., Doneus M., Studnicka N., Rigel J. Combined high resolution laser scanning and photogrammetrical documentation of the pyramids at Giz. Proceedings of the CIPA XX International Symposium.

[11] Sun W., Bradley C., Zhang Y.F., Loh H.T. (2001). Cloud data modelling employing a unified, non-redundant triangular mesh. Comput. Aided Des..

[12] Suveg I., Vosselman G. (2002). Automatic 3D building reconstruction. Three-Dimensional Image Capture and Applications V. Int. Soc. Opt. Photonics.

[13] Besl P.J., Neil D.M. (1992). Method for registering of 3D shapes. IEEE Trans. Pattern Anal. Mach. Intell..

[14] Li Q., Griffiths J.G. (2000). Iterative closest geometric objects registering. Comput. Math. Appl..

[15] Chen H., Bhanu B. Contour registering for 3D ear recognition. Proceedings of the IEEE Workshop on Application of Computer Vision.

[16] Choi S.I., Park S.Y., Kim J. Multi-view range image registering using CUDA. Proceedings of the International Technical Conference on Circuits Systems, Computers and Communications.

[17] He S.J., Zhao S.T., Bai F., Wei J. (2013). A method for spatial data registration based on PCA-ICP algorithm. Adv. Mater. Res..

[18] Yang J., Li H., Jia Y. Go-icp: Solving 3d registration efficiently and globally optimally. Proceedings of the IEEE International Conference on Computer Vision.

[19] Sharp G.C., Lee S.W., Wehe D.K. (2002). ICP registration using invariant features. IEEE Trans. Pattern Anal. Mach. Intell..

[20] Biber P., Strasser W. The normal distributions transform: A new approach to laser scan registering. Proceedings of the 2003 IEEE /RJS International Conference on Intelligent Robots and Systems.

[21] Wen W., Hsu L.T., Zhang G. (2018). Performance analysis of NDT-based graph SLAM for autonomous vehicle in diverse typical driving scenarios of Hong Kong. Sensors.

[22] Sobreira H., Costa C.M., Sousa I., Rocha L. (2019). Map-matching algorithms for robot self-localization: A comparison between perfect match, iterative closest point and normal distributions transform. J. Intell. Robot. Syst..

[23] Pang S., Kent D., Cai X., Al-Quassab H., Morris D., Radha H. 3d scan registration based localization for autonomous vehicles-a comparison of ndt and icp under realistic conditions. Proceedings of the IEEE 88th Vehicular Technology Conference (VTC-Fall).

[24] Attia M., Slama Y., Peyrodie L., Cao H., Haddad F. 3D Point Cloud Coarse Registration based on Convex Hull Refined by ICP and NDT. Proceedings of the 25th International Conference on Mechatronics and Machine Vision in Practice M2VIP.

[25] Wang Q., Zhang J. Point Cloud Registration Algorithm Based on Combination of NDT and ICP. Proceedings of the 2019 15th International Conference on Computational Intelligence and Security (CIS).

[26] Guiyang Z., Zhuang Y., Gang T. (2019). A Point Cloud Registration Method Based on NDT and ICP Fusion. Beijing Surv. Mapp..

[27] Yanming W., Xiaolong Z., Yunfei X. (2020). Research on automatic measurement technology of 3D topography based on NDT-ICP. Mech. Electr. Inf..

[28] Shang Y., Han Z., Qiao Y., Zhou J. (2020). Visualization analysis of the journal of intelligent & fuzzy systems (2002–2018). J. Intell. Fuzzy Syst..

[29] Xiankun L., Longgao L., Dong L. (2018). Experimental analysis on measurement accuracy of lidar. Mech. Manuf. Autom..

[30] Lowe D.G. (2004). Distinctive image features from scale-invariant keypoints. Int. J. Comput. Vis..

[31] Weiser M., Deuflhard P., Erdmann B. (2007). Affine conjugate adaptive Newton methods for nonlinear elastomechanics. Optim. Methods Softw..

[32] Magnusson M., Lilienthal A., Duckett T. (2007). Scan registering for autonomous mining vehicles using 3D-NDT. J. Field Robot..

